# Effects of previous exposure to psychotherapeutic strategies on depression and anxiety symptoms during the COVID-19 pandemic

**DOI:** 10.1192/bjo.2020.170

**Published:** 2021-01-19

**Authors:** Amelia Gulliver, Michelle Banfield, Philip J. Batterham, Alison L. Calear, Louise M. Farrer, Amy Dawel, Sonia McCallum, Kristen Murray, Alyssa R. Morse

**Affiliations:** Centre for Mental Health Research, Research School of Population Health, The Australian National University, Australian Capital Territory, Australia; Centre for Mental Health Research, Research School of Population Health, The Australian National University, Australian Capital Territory, Australia; Centre for Mental Health Research, Research School of Population Health, The Australian National University, Australian Capital Territory, Australia; Centre for Mental Health Research, Research School of Population Health, The Australian National University, Australian Capital Territory, Australia; Centre for Mental Health Research, Research School of Population Health, The Australian National University, Australian Capital Territory, Australia; Research School of Psychology, The Australian National University, Australian Capital Territory, Australia; Centre for Mental Health Research, Research School of Population Health, The Australian National University, Australian Capital Territory, Australia; Research School of Psychology, The Australian National University, Australian Capital Territory, Australia; Centre for Mental Health Research, Research School of Population Health, The Australian National University, Australian Capital Territory, Australia

**Keywords:** Psychotherapeutic, anxiety, depression, COVID-19, psychotherapy

## Abstract

**Background:**

The COVID-19 pandemic has seen an increase in depression and anxiety among those with and without a history of mental illness. Commonly used forms of psychological therapy improve mental health by teaching psychotherapeutic strategies that assist people to better manage their symptoms and cope with life stressors. Minimal research to date has explored their application or value in managing mental health during significant broad-scale public health crises.

**Aims:**

To determine which psychotherapeutic strategies people who have previously received therapy use to manage their distress during the COVID-19 pandemic, and whether the use and perceived helpfulness of these strategies has an effect on symptoms of depression and anxiety.

**Method:**

Data (*N* = 857) was drawn from multiple waves of a representative longitudinal study of the effects of COVID-19 on the mental health of Australian adults, which includes measures of anxiety, depression and experiences with psychotherapy and psychotherapeutic strategies.

**Results:**

Previous engagement in therapy with psychotherapeutic strategies had a protective effect on depressive but not anxiety symptoms. Common and helpful strategies used by respondents were exercise, mindfulness and breathing exercises. Using mindfulness and perceiving it to be helpful was associated with lower levels of depression and anxiety symptoms. No other strategies were associated with improved mental health.

**Conclusions:**

Prior knowledge of psychotherapeutic strategies may play a role in managing mental health during unprecedented public health events such as a global pandemic. There may be value in promoting these techniques more widely in the community to manage general distress during such times.

The COVID-19 pandemic has had significant effects on a global scale, with the number of cases and deaths growing rapidly worldwide.^1^ The World Health Organization has recommended various public health measures, including physical distancing of people to slow the transmission of the coronavirus (SARS-CoV-2) that causes COVID-19.^2^ Accordingly, strict physical distancing measures were enacted in many countries, to varying degrees. In Australia, these ‘lockdown’ measures included requiring people to stay at home unless shopping for essentials such as food or medicines, exercising, going to work if this could not be done from home, or providing care or attending healthcare; limiting non-essential gatherings of people (except households) to no more than two people; maintaining a distance of at least 1.5 m between people when conducting essential activities; and the closure of many businesses where people gather in larger groups, including restaurants, bars, cinemas, gyms and public attractions.^3^ Repeated lockdowns continued both within Australia, and in countries across the world, to regain control over transmission. However, little is known worldwide about the effects of these strategies on people in the community, with growing concerns about the implications of these practices for mental health.^4,5^ A cross-sectional and national, although not necessarily representative, USA online survey (*N* = 500) conducted using during the early stages of the pandemic (March 27 to April 5 2020) indicated that stay-at-home orders were associated with health anxiety, financial worry and loneliness.^6^ In addition, mental health during an unprecedented event of this scale can also be negatively affected by other distressing factors, such as financial concerns, job loss^7^ and worry about contracting the illness.^6,8^ Nationally representative research is needed;^9,10^ however, there are few representative studies, such as that conducted by Pierce et al,^11^ investigating the effects of the pandemic and associated physical distancing restrictions implemented to contain it on mental health. Many studies (e.g. Wang et al^12^) have used convenience or snowball sampling techniques, and the role of protective factors has been underresearched when compared with risk factors.

## The effect of previous mental illness

Mental disorders such as depression and anxiety are common.^13^ There is now robust evidence from representative, community-based longitudinal surveys that symptoms of these disorders have been elevated during the pandemic.^11,14^ Cross-sectional survey research has also shown that people with existing mental health problems before the start of the COVID-19 pandemic may experience an exacerbation of their illness.^15^ However, it is unclear how these individuals have been coping. Thus, it is vital to investigate the experience of people who have had previous treatment that taught practical skills and strategies (‘psychotherapeutic strategies’), as this experience may buffer the effects of significant crises, such as a pandemic, on their mental health. Given expected rises in symptoms of common mental health problems such as depression and anxiety, even in those without a history of diagnosed disorders,^14^ it is critically important to consider how we can best support people, especially those with pre-existing mental health conditions, during such a time.

## The role of psychological therapies

The type of therapy an individual receives varies depending on many factors, including the type of psychological problem or disorder, therapist skill and availability, and personal preference.^16,17^ Mental health problems such as depression and anxiety are commonly treated with psychotherapies that have a strong evidence base, such as cognitive–behavioural therapy;^18^ are standardised and have high fidelity; and involve the use of psychotherapeutic strategies, such as pleasant event scheduling^19^ or mindfulness techniques.^20^ These strategies can be used to reduce the chance of disorder relapse^21^ and improve coping with major life stressors.^22^ There has been little previous research into the use or value of psychotherapeutic strategies during global public health crises, such as pandemics, to manage mental health. Previous research conducted during the 2009 H1N1 influenza pandemic^23^ indicated that certain types of strategies, particularly those that were focused on problem-solving (e.g. cognitive restructuring, active distraction), were more helpful at reducing anxiety around the pandemic than those that focused more on controlling emotions (e.g. wishful thinking, emotional expression). Innovative treatment programmes are now focused on examining the efficacy of specific strategies with a focused theoretical aim, such as cognitive restructuring or exposure techniques, rather than examining the collective effect of a treatment programme in which a range of strategies may be employed.^24^ This allows an evaluation of the specific techniques or skills learned in isolation. However, very little is known about the effects of learning specific psychotherapeutic strategies on people's ability to cope during a large-scale crisis. Thus, the aim of this paper is to examine the potentially protective effect of prior exposure to psychotherapeutic strategies on symptoms of depression and anxiety during the COVID-19 pandemic. Specifically, we sought to investigate (a) if learning psychotherapeutic strategies through previous therapy was associated with lower symptoms of depression or anxiety, contingent on past or current mental health diagnosis; (b) what specific psychotherapeutic strategies people who had previously engaged in therapy found most useful in managing their distress during the pandemic; and (c) if the actual application and perceived helpfulness of specific psychotherapeutic strategies during the pandemic, among people who previously received psychosocial therapy that taught practical strategies, was associated with lower symptoms of depression or anxiety.

## Method

### Ethics approval

The authors assert that all procedures contributing to this work comply with the ethical standards of the relevant national and institutional committees on human experimentation and with the Helsinki Declaration of 1975, as revised in 2008. All procedures involving human participants were approved by The Australian National University Human Research Ethics Committee (protocol number 2020/152).

### Study design

‘The Australian National COVID-19 Mental Health, Behaviour and Risk Communication (COVID-MHBRC) Survey’ was designed to investigate the mental health effects of the COVID-19 pandemic on a representative sample of Australian adults (≥18 years). The survey used a longitudinal design and comprised seven waves of data collected fortnightly, using the Qualtrics Research Services online platform. Surveys remained open for completion for 7 days after each launch date.

### Recruitment

Participants were required to be living in Australia, and able to respond to an online English language survey. To enable the collection of a representative sample, recruitment was conducted by quota sampling, using categories of age group, gender and geographic location (Australian state/territory). Informed consent was obtained from all participants. Participants read an information sheet explaining the study, and provided their informed consent by clicking ‘yes’ that they agreed to commence the survey. Those who clicked ‘no’ were taken to a thank you page. The full study protocol is available online (https://psychology.anu.edu.au/files/COVID_MHBRCS_protocol.pdf).

### Participants

The baseline data-set included 1296 participants (645 males, 649 females, 2 missing data on gender). The sample size required was based on power analyses for linear regression models, setting Beta at (1 − *β)* = 0.95 and *α* = 0.05, considering both attrition over waves and an allowance for erroneous data. We report data from participants (*n* = 857; 425 males, 422 females) who completed assessment at both wave 3, in which measures of exposure to and use of psychotherapeutic strategies were collected (launched 25 April 2020), and wave 4 (launched 9 May 2020).

### Survey measures

#### Demographic characteristics

The following demographic characteristics were collected at the baseline (wave 1) assessment: age (in years), gender (male/female/other), level of education (high school or less, certificate/diploma, bachelor's degree, higher degree), partner status (categorised as yes/no), living alone (yes/no), living with dependent children (yes/no) and experience of a mental disorder as diagnosed by an appropriate clinician (e.g. general practitioner, psychiatrist, psychologist) (yes/no). We also collected a range of other variables in each wave,^14^ which are not included in the current analysis. The variables collected at each wave of the full study are available online (https://psychology.anu.edu.au/files/20200702_CovidMHBRCS_waveContent_FINAL.pdf).

#### Symptoms of anxiety and depression

The Patient Health Questionnaire-9 (PHQ-9)^23^ was used to assess depression symptoms. The PHQ-9 comprises nine items that assess the frequency of DSM-IV symptoms of major depression during the past 2 weeks. Items are rated on a four-point scale, ranging from not at all (0) to nearly every day (3), and are summed to produce an overall severity score (range 0–27). Higher scores indicate higher depression symptom severity. The PHQ-9 has shown good sensitivity (0.77–0.88) and specificity (0.88–0.94) for detecting major depression in clinical and general population samples,^25^ and has acceptable internal consistency in the general population (*α* = 0.87),^26^ which was similar in this sample for the data we used, which was collected at wave 4 (*α* = 0.93).

The Generalized Anxiety Disorder-7 (GAD-7) was used to assess anxiety symptoms. The GAD-7 scale comprises seven items that correspond to DSM-IV and DSM-5 criteria for generalised anxiety disorder (GAD).^27^ Items are rated on the same four-point scale as the PHQ-9. Scores for each item are summed (range 0–21), and higher scores indicate greater symptom severity. Previous research has demonstrated that the GAD-7 has good psychometric properties in general population and clinical samples (acceptable internal consistency of *α* = 0.89–0.92^27,28^), and provides accuracy compared with clinical diagnosis.^25,28^ The internal consistency was good in this sample for the data we used, which was collected at wave 4 (*α* = 0.94).

#### Experience with psychotherapy and psychotherapeutic strategies

Four items were used to assess participants’ prior experience with psychological therapy and psychotherapeutic strategies. The first question asked: ‘Before the COVID-19 pandemic, what was your experience with psychological therapy?’. Response options were ‘None, I never received therapy before COVID-19’; ‘I received therapy and learned some practical strategies to improve my mental health’; and ‘I received therapy, but did not learn any practical strategies to improve my mental health’. Participants who indicated they learned practical strategies were then asked (a) whether they had used each of a list of ten specific psychotherapeutic strategies during the pandemic and, if so, how helpful they had found that strategy for managing their mental health (ranging from 1, not at all helpful, to 4, very helpful); (b) to what extent had they used these strategies during the pandemic (ranging from 1, not at all, to 4, quite a lot); and (c) to what extent they believed these strategies had helped them cope during the pandemic (ranging from 1, not at all, to 4, quite a lot). An ‘other (please specify)’ option was also provided to allow respondents to list and rate a strategy not listed. This category was not formally explored; however, participants (*n* = 24) who responded noted a range of broad strategies, such as meditation or prayer, music, relaxing and talking to friends/family. The ten psychotherapeutic strategies listed were mindfulness, breathing exercises, progressive muscle relaxation, challenging dysfunctional thoughts, thought records, scheduling pleasant events, planning ‘worry’ time, flash cards, using a diary and exercise. Supplementary File 1 available at https://doi.org/10.1192/bjo.2020.170 provides descriptions of the psychotherapeutic strategies, which were generated by the lead author searching relevant literature and identifying commonly used techniques in therapy.

### Analyses

We expected that participants may be using psychotherapeutic strategies to cope during this time. To capture the impact of these strategies on mental health, we used two consecutive waves of data specifically to assess effects of the use of these strategies on subsequent symptoms of depression and anxiety in the shortest possible time frame. The time between waves for the full longitudinal survey was deliberately brief (2 weeks), to capture any fluctuations that may have occurred during this rapidly evolving public health crisis. We used two consecutive waves of data because this study was explicitly designed to examine the effect of strategies on subsequent mental health, rather than concurrent or retrospective mental health outcomes. By doing this, we were able to provide stronger evidence related to the direction of the effects of the use of psychotherapeutic strategies on symptoms of depression and anxiety.

We used multiple waves of data from the survey to rigorously assess if specific factors identified at wave 1 (demographic data from late March 2020) and wave 3 (use of psychological therapies and strategies reported in late April 2020) predicted symptoms of depression and anxiety at wave 4 (mid-May 2020). There were large correlations between mental health measures at wave 3 and wave 4 for both PHQ-9 depression (*r* = 0.82) and GAD-7 anxiety (*r* = 0.82) symptoms. Because of these strong associations and our focus on level rather than change in symptom levels, wave 3 scores were not included in the regression models. To test whether prior engagement in therapy with psychotherapeutic strategies was associated with symptoms of depression and anxiety, two linear regression analyses were estimated to identify the mental health history-related wave 3 factors that were associated with depression (PHQ-9) and anxiety (GAD-7) symptoms at wave 4. Independent variables included in these models were past use of therapy (no, yes/did not learn strategies, yes/did learn strategies), history of mental health diagnosis (self-reported; never, past, current), gender, level of education, age and the two-way interaction between use of therapy and history of mental health diagnosis. Descriptive statistics were used to explore the second aim, identifying which psychotherapeutic strategies were most commonly used and which strategies were reported to be most helpful. The third aim, whether these strategies resulted in lower depression and anxiety symptoms, was tested by two additional linear regression analyses, assessing associations between strategies used at wave 3 with symptoms of depression (PHQ-9) and anxiety (GAD-7) at wave 4, among the subsample of individuals who had used strategies from therapy to manage their mental health during the COVID-19 pandemic. These analyses were also adjusted for age, gender and education. Statistical tests were conducted with SPSS version 26 for Windows (IBM Corp., Chicago, IL).

## Results

From the sample of 857 participants, 256 (30%) reported receiving previous therapy, of whom 195 (76%) reported learning strategies and 61 (24%) reported learning no strategies. Table 1 reports the current study sample distributions by exposure to psychotherapy. The demographic distributions were similar to the original sample,^14^ which aligned well with population data from the Australian Bureau of Statistics, indicating it was a representative sample of the Australian community. *χ*^2^ and one-way ANOVA were used to identify statistical differences between the therapy groups. Follow-up *t*-tests and *χ*^2^-tests were used to identify where the differences occurred between groups.

**Table 1:** Sample characteristics by exposure to previous psychotherapy

	Full sample, waves 3 and 4 (*N* = 857)^a^	Therapy with strategies (*n* = 195; 22.8%)	Therapy with no strategies (*n* = 61; 7.1%)	No therapy (*n* = 599; 69.9%)
Demographics (wave 1)				
Age, years, mean (s.d.)*	50.02 (16.14)	47.01 (15.39)	49.38 (17.42)	51.10^b^ (16.15)
Male, *n* (%)	435 (50.8)	86 (44.1)	31 (50.8)	317 (52.9)
Female, *n* (%)	422 (49.2)	109 (55.9)	30 (49.2)	282 (47.1)
Current mental disorder, *n* (%)**	200 (23.3)	98 (50.3)	32 (52.5)	69 (11.5)^c,d^
Past mental disorder, *n* (%)**	159 (18.6)	56 (28.7)	13 (21.3)	89 (14.9)^c^
Never diagnosed, *n* (%)**	498 (23.3)	41 (21.0)	16 (26.2)	441 (73.6)^c,d^
Education (wave 1)				
Certificate/diploma	319 (37.2)	78 (40.0)	27 (44.3)	214 (35.7)
Bachelor's degree	262 (30.6)	61 (31.3)	19 (31.1)	181 (30.2)
Higher degree	39 (4.6)	8 (4.1)	2 (3.3)	29 (4.8)
High school or less	233 (27.2)	46 (23.6)	13 (21.3)	173 (28.9)
No response	4 (0.5)	2 (1.0)	0 (0)	2 (0.3)
Mental health measures (wave 3/4)				
Wave 3 PHQ-9, mean (s.d.)**	5.53 (6.01)	7.46 (6.22)	9.89^e^ (7.74)	4.46^f,g^ (5.38)
Wave 4 PHQ-9, mean (s.d.)**	5.16 (5.83)	7.13 (5.93)	9.64^b^ (7.71)	4.05^f,g^ (5.16)
Wave 3 GAD-7, mean (s.d.)**	4.39 (5.14)	6.22 (5.47)	7.84 (6.24)	3.44^f,g^ (4.57)
Wave 4 GAD-7, mean (s.d.)**	4.05 (4.94)	5.72 (5.18)	7.57^e^ (6.30)	3.13^f,g^ (4.38)

a. Two participants did not complete the initial question about previous therapy experience, but completed subsequent sections on their use of therapy strategies; thus their data is included in the full sample (*N* = 857) characteristics only. Data from these two participants is included in the summary table (Table 3).

b. *t*-test indicated a significant difference versus therapy with strategies, *p* < 0.01.

c. χ^2^ indicated a significant difference versus therapy with strategies, *p* < 0.001.

d. χ^2^ indicated a significant difference versus therapy with no strategies, *p* < 0.001.

e. *t*-test indicated a significant difference versus therapy with strategies, *p* < 0.05.

f. *t*-test indicated a significant difference versus therapy with strategies, *p* < 0.001.

g. *t*-test indicated a significant difference versus therapy with no strategies, *p* < 0.001.

* *p* < 0.05 One-way ANOVA indicated a significant difference between therapy groups.

** *p* < 0.001 χ^2^ or one-way ANOVA indicated a significant difference between therapy groups.

### Effects of therapy on symptoms of depression and anxiety

Table 2 presents the linear regression models for depression and anxiety symptoms, using the complete sample that had data from both waves 3 and 4 (*N* = 857). The estimates indicated that exposure to therapy both with or without strategies was associated with more severe symptoms of depression and anxiety compared with those who had experienced no therapy. Individuals who learned strategies from therapy had less severe (lower) symptoms than those who reported learning no strategies. Not surprisingly, past or current diagnosis was associated with significantly more severe depression and anxiety symptoms. Younger age was also associated with more severe symptoms, although gender and education were not. The interaction between diagnosis and use of therapy was significant for both depression and anxiety. The interaction demonstrated that people with a current or past mental health diagnosis had lower symptoms of depression if they had engaged in therapy with psychotherapeutic strategies, whereas those with a past but not current diagnosis also had lower symptoms of depression if they engaged in therapy without learning any strategies. For those with a current diagnosis, engaging in therapy without learning strategies was not protective against depression symptoms. A different pattern emerged for anxiety symptoms. Therapy was only associated with lower anxiety symptoms among those with a past diagnosis who had not learned strategies. Therapy did not appear to be associated with lower levels of symptoms among individuals with a current diagnosis.

**Table 2:** Linear regression models for depression (PHQ-9) and anxiety (GAD-7) at Wave 4 (W4) predicted by demographic data (*N* = 857)

	PHQ-9 (wave 4)				GAD-7 (wave 4)			
	Estimate	s.e.	*t/F*	*P*-value	Estimate	s.e.	*t*	*P-*value
Intercept	7.778	0.764	10.175	<0.001	6.247	0.661	9.451	<0.001
Exposure to therapy (wave 3)			5.145	**0.006**			5.226	**0.006**
Therapy, learned strategies	2.444	0.823	2.971	**0.003**	1.622	0.711	2.280	**0.023**
Therapy, no strategies	3.827	1.271	3.012	**0.003**	3.670	1.099	3.340	**0.001**
No therapy	(Reference)				(Reference)			
History of mental health diagnosis (wave 1)			36.556	**<0.001**			29.579	**<0.001**
Past	2.713	0.581	4.666	**<0.001**	1.849	0.503	3.679	**<0.001**
Current	5.062	0.648	7.808	**<0.001**	3.913	0.561	6.982	**<0.001**
Never	(Reference)				(Reference)			
Female versus male gender (wave 1)	0.053	0.355	0.149	0.881	0.108	0.307	0.352	0.725
Education (wave 1)			0.801	0.525			0.738	0.566
Certificate/diploma	0.283	0.433	0.653	0.514	0.333	0.374	0.890	0.374
Bachelor's degree	0.106	0.465	0.227	0.821	0.060	0.402	0.150	0.881
Higher degree	−1.014	0.869	−1.167	0.244	−0.728	0.752	−0.968	0.333
Refused	−2.110	2.525	−0.836	0.404	−1.382	2.183	−0.633	0.527
High school or less	(Reference)				(Reference)			
Age (wave 1)	−0.094	0.011	−8.346	**<0.001**	−0.078	0.010	−7.999	**<0.001**
Therapy (wave 3) × history (wave 1)			4.797	**0.001**			3.136	**0.014**
Therapy/learned × past	−3.347	1.179	−2.839	**0.005**	−1.898	1.019	−1.862	0.063
Therapy/learned × current	−2.275	1.140	−1.996	**0.046**	−1.207	0.986	−1.224	0.221
Therapy/no strategies × past	−5.221	1.959	−2.665	**0.008**	−4.715	1.694	−2.783	**0.006**
Therapy/no strategies × current	0.799	1.659	0.481	0.630	−0.232	1.435	−0.162	0.872

Significant results are indicated in bold. PHQ-9, Patient Health Questionnaire-9; GAD-7, Generalized Anxiety Disorder-7 scale.

### Use and perceived helpfulness of psychological strategies

All 195 (100%) participants who reported receiving therapy with psychotherapeutic strategies reported they had tried at least one of the strategies to manage their mental health during the COVID-19 pandemic. However, when specifically asked about their use of strategies, a very small percentage (7.1%) said they had not used the strategies at all. Table 3 shows that the most common strategies used by more than three-quarters of participants were exercise, breathing exercises, mindfulness and challenging dysfunctional thoughts. Less than a third had used flash cards. On average, all strategies (except flash cards) were rated as being at least a little helpful, with the most helpful strategies overall reported to be exercise, mindfulness, breathing exercises and pleasant events scheduling. Over half of participants had somewhat used these strategies, and a similar proportion found the strategies somewhat to very helpful in assisting them to cope during the COVID-19 pandemic.

**Table 3:** Perceived helpfulness of psychotherapeutic strategies (*n* = 197)

	Used strategy	Not used	Very helpful (4)	Somewhat helpful (3)	A little helpful (2)	Not at all helpful (1)	Helpfulness rating (range 1–4)^b^	Total *N*^c^
	*N* (%)		*n* (%)^a^	*n* (%)	*n* (%)	*n* (%)	Mean (s.d.)	
Strategy								
Exercise	180 (92.3)	15 (7.7)	67 (37.2)	53 (29.4)	50 (27.8)	10 (5.6)	2.98 (0.93)	195
Breathing exercises	159 (80.7)	38 (19.3)	31 (19.5)	60 (37.7)	54 (34.0)	14 (8.8)	2.68 (0.89)	197
Pleasant events scheduling	133 (68.2)	62 (31.8)	22 (16.5)	52 (39.1)	54 (40.6)	5 (3.8)	2.68 (0.79)	195
Challenging dysfunctional thoughts	147 (75.0)	49 (25.0)	27 (18.4)	42 (28.6)	61 (41.5)	17 (11.6)	2.53 (0.92)	196
Progressive muscle relaxation	128 (65.0)	69 (35.0)	21 (16.4)	54 (42.2)	43 (36.6)	10 (7.8)	2.67 (0.84)	197
Mindfulness	161 (81.7)	36 (18.3)	26 (16.1)	66 (41.0)	63 (39.1)	6 (3.7)	2.71 (0.78)	197
Using a diary	83 (42.3)	113 (57.7)	12 (14.5)	27 (32.5)	28 (33.7)	16 (19.2)	2.42 (0.96)	196
Thought records	99 (50.3)	98 (49.7)	10 (10.1)	32 (32.3)	46 (46.5)	11 (11.1)	2.41 (0.82)	197
Planning ‘worry’ time	79 (40.1)	118 (59.9)	5 (6.3)	19 (24.0)	30 (38.0)	25 (31.7)	2.05 (0.90)	197
Flash cards	60 (30.5)	137 (69.5)	2 (3.3)	11 (18.3)	21 (35.0)	26 (43.3)	1.82 (0.85)	197
			Quite a lot (4)	Somewhat (3)	A little (2)	Not at all (1)	Rating	
			*n* (%)	*n* (%)	*n* (%)	*n* (%)	(range 1–4)	
To what extent did you use these strategies?	–	–	24 (12.2)	80 (40.6)	79 (40.1)	14 (7.1)	2.58 (0.80)	197
To what extent do you believe they helped you to cope?	–	–	38 (19.3)	73 (37.1)	70 (35.5)	16 (8.1)	2.68 (0.88)	197

a. Percentage of helpfulness calculated from those who used the strategy.

b. Data from those who used the strategy only.

c. Total *N* varies as not all participants provided data for every item.

### Associations between use and perceived helpfulness of specific psychotherapeutic strategies and symptoms of depression and anxiety

Table 4 presents the linear regression models for the effect of specific strategies on depression and anxiety symptoms, using the subsample of those who completed waves 3 and 4, reported receiving therapy with psychotherapeutic strategies and had complete data on their use of strategies *(n* *=* 192). The interaction between the technique of mindfulness and its perceived usefulness was significant for both depression and anxiety. The interaction demonstrated that people who had used mindfulness and perceived it as useful had lower symptoms of depression and anxiety. However, the reverse was true for breathing exercises, where those who had used breathing exercises and perceived them as useful had higher symptoms of depression and anxiety. This was also the case for flash cards, where those who perceived flash cards as useful had significantly lower depression scores at wave 4.

**Table 4:** Linear regression models for depression (PHQ-9) and anxiety (GAD-7) at wave 4, predicted by use of strategies and their perceived helpfulness (*n* = 192)

	PHQ-9 (wave 4)				GAD-7 (wave 4)			
	Estimate	s.e.	*t*	*P-*value	Estimate	s.e.	*t*	*P-*value
Intercept	14.963	2.519	5.939	(Reference)	10.475	2.233	4.690	(Reference)
Mindfulness								
Used/not helpful	−0.773	1.383	−0.559	0.577	−1.297	1.226	−1.058	0.292
Used/helpful	−3.564	1.450	−2.458	**0.015**	−3.420	1.286	−2.660	**0.009**
Did not use	(Reference)				(Reference)			
Breathing exercises								
Used/not helpful	0.657	1.367	0.481	0.631	0.422	1.212	0.349	0.728
Used/helpful	3.377	1.542	2.191	**0.030**	3.364	1.367	2.461	**0.015**
Did not use	(Reference)				(Reference)			
Progressive muscle relaxation								
Used/not helpful	1.958	1.299	1.507	0.134	1.742	1.152	1.512	0.132
Used/helpful	0.533	1.387	0.384	0.701	1.045	1.230	0.850	0.397
Did not use	(Reference)				(Reference)			
Challenging dysfunctional thoughts								
Used/not helpful	−1.339	1.213	−1.104	0.271	−0.017	1.076	−0.016	0.987
Used/helpful	−0.536	1.344	−0.399	0.690	−0.825	1.191	−0.693	0.489
Did not use	(Reference)				(Reference)			
Thought records								
Used/not helpful	0.941	1.311	0.718	0.474	1.897	1.162	1.632	0.105
Used/helpful	−0.917	1.563	−0.587	0.558	−0.219	1.386	−0.158	0.875
Did not use	(Reference)				(Reference)			
Pleasant events scheduling								
Used/not helpful	1.687	1.194	1.413	0.160	1.637	1.059	1.546	0.124
Used/helpful	1.088	1.276	0.853	0.395	0.938	1.132	0.829	0.408
Did not use	(Reference)				(Reference)			
Planning ‘worry’ time								
Used/not helpful	1.404	1.633	0.860	0.391	0.171	1.448	0.118	0.906
Used/helpful	−0.935	2.207	−0.424	0.672	0.481	1.957	0.246	0.806
Did not use	(Reference)				(Reference)			
Flash cards								
Used/not helpful	−0.450	1.706	−0.264	0.792	−1.198	1.513	−0.792	0.429
Used/helpful	5.478	2.540	2.157	**0.032**	2.564	2.251	1.139	0.256
Did not use	(Reference)				(Reference)			
Using a diary								
Used/not helpful	−0.749	1.423	−0.526	0.599	−0.819	1.262	−0.649	0.517
Used/helpful	−1.156	1.399	−0.826	0.410	−0.670	1.240	−0.540	0.590
Did not use	(Reference)				(Reference)			
Exercise								
Used/not helpful	0.758	1.690	0.448	0.655	0.939	1.499	0.627	0.532
Used/helpful	−1.430	1.619	−0.884	0.378	−0.574	1.435	−0.400	0.690
Did not use	(Reference)				(Reference)			
Gender								
Female	−0.898	0.886	−1.013	0.312	−0.478	0.786	−0.609	0.544
Male	(Reference)				(Reference)			
Education								
Certificate/diploma	0.331	1.064	0.312	0.756	−0.522	0.943	−0.553	0.581
Bachelor's degree	−1.180	1.111	−1.062	0.290	−1.398	0.985	−1.419	0.158
Higher degree	0.335	2.379	0.141	0.888	−0.977	2.109	−0.463	0.644
No response	−5.597	4.014	−1.394	0.165	−6.308	3.558	−1.773	0.078
High school or less	(Reference)				(Reference)			
Age, years	−0.159	0.030	−5.254	**<0.001**	−0.106	0.027	−3.959	**<0.001**

Significant results are indicated in bold. PHQ-9, Patient Health Questionnaire-9; GAD-7, Generalized Anxiety Disorder-7 scale.

## Discussion

Overall, almost all participants who reported any previous experience of therapy using psychotherapeutic strategies reported using those strategies to cope during the COVID-19 pandemic. The effects for therapy with and without psychotherapeutic strategies on depression and anxiety symptoms was mixed. For depressive symptoms, those with a current or past mental health diagnosis had lower levels of symptoms if they had exposure to therapy reported to include training in psychotherapeutic strategies. However, only those with a past (but not current) diagnosis had lower symptoms of depression if they had exposure to therapy without learning such strategies. For anxiety, only those with a past diagnosis who had previously had therapy without learning strategies had lower anxiety symptoms. Additionally, those who reported using the strategy of mindfulness and perceived it to be helpful had significantly lower levels of depression and anxiety symptoms. Finally, those who used and perceived breathing exercises to be helpful had higher levels of depression and anxiety, and the use of flash cards, although perceived as helpful, was associated with higher levels of depression.

For almost all psychotherapeutic strategies (eight out of ten), four out of five people who had used the strategies perceived them to be at least a little helpful in coping. The most useful strategy overall was perceived to be exercise, which is currently recommended as an evidence-based strategy for improving mental health problems, including depression and anxiety.^29,30^ It is also recommended as being particularly useful as an adjunct to treatments such as antidepressants.^29^ Other frequently used strategies that were perceived by people to be helpful included mindfulness, pleasant events scheduling, progressive muscle relaxation, challenging dysfunctional thoughts and breathing exercises. It is possible that the most frequently used strategies demonstrate the kinds of therapeutic strategies that are likely to be remembered, and more easily applied, by people in times of stress. In addition, people may be more likely to remember and use the strategies they find subjectively helpful. It is also important to recognise that certain strategies may have been particularly appropriate in the context of a global public health event such as the COVID-19 pandemic. Although we did not ask where people exercised, given the context of the lockdown, it is possible that some may have frequently used and found exercise helpful because it was an allowable reason to leave the confines of their home. They could experience fresh air while exercising, and this may have also included the bonus of a degree of social interaction from a distance. Moreover, deliberately carving out time to engage in a pleasant and enjoyable event also makes sense in this context, particularly as many social and commercial activities were halted. These benefits suggest that rates of exercise were likely high in the general community during this time, regardless of therapy exposure. The strategy of mindfulness could also have been useful during this time, as the experience of focusing on and appreciating the present in a time of great uncertainty about the future may have been helpful for people. In comparison, cognitive strategies, such as challenging dysfunctional thoughts, may be more difficult in a time of significant uncertainty. Thus, the nature of the context surrounding a significant public health event, as well as the strategies individuals prefer and find personally effective,^31^ may be relevant in determining which strategies to recommend. Moreover, being able to select from a range of preferred strategies that can be employed in a crisis is likely to be helpful for people.

The estimates indicated that exposure to therapy overall was associated with more severe symptoms of depression and anxiety, which is to be expected as individuals are more likely to seek help if they have greater need.^32^ However, the relationship between diagnosis and therapy type was quite complex, suggesting important differences in the supports people need in times of crisis. Findings for depression were as expected, with practical strategies associated with lower scores, but therapy with practical strategies was not associated with lower anxiety. There are several possible explanations for this. The relationship between the perceived benefits of past therapy and symptom levels is likely to be complex, with factors such as satisfaction with the therapist, time since therapy, type of therapy received and its duration, and mental health characteristics mediating the relationship. The complexity of symptoms of depression and anxiety in terms of comorbidity, severity and potential exacerbation because of the diverse effects of the COVID-19 pandemic may also explain the heterogeneity of the observed relationships. Although we were able to prospectively examine the effects of perceived therapy benefits on mental health outcomes over a 2-week period, there are likely to remain issues related to reverse causation and the variable time course of treatment effects that limit the consistency of findings.

Higher levels of anxiety and depression symptoms were found in participants who found breathing techniques helpful during COVID-19. The regulation of breathing is a treatment that can be used for anxiety,^33^ which can help to correct problematic breathing patterns, although its effectiveness is inconsistent and may be limited to panic disorder.^34^ The participants’ view that this technique was helpful may have been because it had previously brought about reduced symptoms, and during COVID-19 it may have enabled participants to feel more in control of their situation. In certain situations, some therapists have viewed breathing techniques as providing therapeutic effects by gaining control rather than addressing breathing irregularities.^34,35^ Thus, it is possible that the breathing techniques may have been used ineffectively or inappropriately. For example, for some people who use this technique, breathing exercises may become a safety behaviour, which then contributes to the anxiety maintenance cycle. This illustrates the disconnect that can occur between symptom-based measures of mental health and the subjective experience of helpfulness. Another possible alternative explanation is that participants who used breathing techniques were aware that those strategies were preventing subsequently worse symptoms had they not used them.

We found the technique of mindfulness was associated with lower anxiety when used by people who perceived it to be helpful. A highly prominent theme in the current pandemic is worry and uncertainty about the future.^36,37^ Previous studies have found that mindfulness mediates the relationship between intolerance of uncertainty and the experience of anxiety and depression.^38^ Thus, this may be why this technique has been viewed as helpful, and was associated with a reduction in symptoms. It is also possible that mindfulness may also have additional benefits, such as increasing introspective awareness of symptoms. Perhaps it may help people enact other supportive strategies and engage in relapse prevention. Mindfulness is a strategy that can be learned through practice. Periods of enforced isolation or ‘lockdown’ may provide opportunity for the broad dissemination of self-guided online training programmes that teach skills and strategies to manage symptoms of common mental health problems. However, there was no effect among participants who did not perceive mindfulness to be helpful, and effects on symptoms among those who did find it helpful were moderate (*d* = 0.61 for depression symptoms, *d* = 0.69 for anxiety symptoms).

### 

#### Limitations

The current study has several limitations. First, we could not control for the highly variable ways in which these techniques might have been used by participants. We did not ask how proficient the participants were in their use of the strategies, which could have been related to their perceived effectiveness. Indeed, there was some divergence between self-reported helpfulness and depression and anxiety symptom change. Second, we did not provide descriptions of what each technique was, and instead relied on people's knowledge of them; for example, flash cards could have been interpreted quite broadly. We also note that the strategies were not intended to be a comprehensive list; they focused primarily on those from commonly used evidence-based therapies, such as cognitive–behavioural therapy. The results may have differed if we had selected different or less commonly used strategies. For example, flash cards were rarely used; it is possible that the significant result regarding the use of flash cards is not statistically reliable, given the small number of people who used flash cards and found them helpful. In addition, although we did attempt to isolate the effects of certain strategies, most are not used in isolation and are usually part of a multicomponent therapeutic approach; thus, it is possible they may not show strong effects when assessed individually. Furthermore, as noted above, we did not collect data on the time since treatment or duration of treatment, and were limited in our ability to account for reverse causation (i.e. higher symptoms of depression or anxiety affecting the individual's selection and use of strategies or their perceived helpfulness). Because we were interested in the effects of therapy and strategies that were learned before the pandemic, we only asked those who had previous exposure to therapy about their use of psychotherapeutic strategies. It is possible that those who had no previous exposure to therapy may have used strategies during this time, although if they had not been taught about strategies and how to use them, they may have been less likely to know how to use them correctly. The effects of psychological strategies may have also interacted with medication use such as antidepressants,^39^ although we did not collect data on participants’ use of antidepressant medication. Finally, although the study was specifically designed to be representative of the Australian population, there may have been some groups who were not represented, such as those without access to the internet.

In conclusion, the results of this study indicate that people use previously learned psychotherapeutic strategies to cope during times of heightened psychological stress and uncertainty, such as the COVID-19 pandemic. Some of these techniques, such as exercise and mindfulness, were perceived to be more helpful at assisting people to cope than others. However, perceived helpfulness was not directly related to lower symptoms for most of the strategies. Further research investigating how perceptions of therapy affect therapeutic outcome during a public health crisis is warranted. Nevertheless, the current study indicates that prior knowledge of psychotherapeutic strategies may play an important role in preventing mental illness in times of crisis, highlighting the need for greater promotion of, and proficiency in, the use of these strategies among the general population.

## Data Availability

The data that support the findings of this study are available from the corresponding author, A.G., upon reasonable request.

## Supplementary material

For supplementary material accompanying this paper visit https://doi.org/10.1192/bjo.2020.170. click here to view supplementary material
