# Supplementary material for: Effects of previous exposure to psychotherapeutic strategies on depression and anxiety symptoms during the COVID-19 pandemic
Source: BJPsych Open. 2021 Jan 19;7(1):e38. doi: 10.1192/bjo.2020.170 (PMC7844171; doi:10.1192/bjo.2020.170)
Supplement: Supplementary file 1 [file S2056472420001702sup001.pdf]

Table S1. List of 10 psychological strategies

Strategy	Definition and examples
Mindfulness (1)	Training and practice that promotes "openly attending, with awareness, to one's present moment experience" (p. 493; (2) E.g., first identify 3 things you can see…then hear…then feel/smell/taste.
Breathing exercises (3)	Training to breathe more deeply, slowly and/or regularly.
Progressive muscle relaxation (4)	Tensing and then relaxing sets of muscles, working systematically across the body (e.g., from feet to head).
Challenging dysfunctional thoughts (5)	Identifying and challenging patterns of thinking that are unhelpful or distorted. E.g., When you identify an instance of jumping-to-conclusions (e.g., "they sounded unhappy, so I must have done something to upset them") generate a list of possible alternative explanations (e.g., "they might be having a bad day").
Thought records (6)	Keeping a record of the thoughts and feelings associated with daily events and behaviours and rating their intensity to identify and challenge dysfunctional thoughts
Scheduling pleasant events (7)	Identifying and scheduling pleasant events to engage in regularly e.g., Schedule time to visit friends, engage in a hobby, eat a nice meal, or take a bath.
Planning "worry" time (8)	Scheduling a block of time each day just for worrying, and then saving worries for this time e.g., When worries come up, make a note of them, but then put them aside for a block of time you've set aside each day for worrying.
Flash cards (9)	Keeping small business sized cards accessible that contain helpful psychotherapeutic strategies, reminders, or statements.
Using a diary (10)	Journaling in a diary using expressive writing, recording a behaviour/thought/emotion of clinical interest in a diary.
Exercise (11, 12).	Engaging in a physical activity that enhances or maintains physical fitness.

Table references

1. Khoury B, Lecomte T, Fortin G, Masse M, Therien P, Bouchard V, et al. Mindfulness-based therapy: a comprehensive meta-analysis. Clin Psychol Rev. 2013;33(6):763-71.
2. Creswell JD. Mindfulness Interventions. Annual Review of Psychology. 2017;68(1):491-516.
3. Jerath R, Crawford MW, Barnes VA, Harden K. Self-Regulation of Breathing as a Primary Treatment for Anxiety. Applied Psychophysiology and Biofeedback. 2015;40(2):107-15.
4. Manzoni GM, Pagnini F, Castelnuovo G, Molinari E. Relaxation training for anxiety: a ten-years systematic review with meta-analysis. BMC Psychiatry. 2008;8(1):41.
5. Lam DCK, Gale J. Cognitive behaviour therapy: an evidence-based clinical framework for working with dysfunctional thoughts. Counselling Psychology Quarterly. 2004;17(1):53-67.

6. McManus F, Van Doorn K, Yiend J. Examining the effects of thought records and behavioral experiments in instigating belief change. Journal of Behavior Therapy and Experimental Psychiatry. 2012;43(1):540-7.
7. Simmonds-Buckley M, Kellett S, Waller G. Acceptability and Efficacy of Group Behavioral Activation for Depression Among Adults: A Meta-Analysis. Behavior Therapy. 2019;50(5):864-85.
8. Borkovec TD, Wilkinson L, Folensbee R, Lerman C. Stimulus control applications to the treatment of worry. Behaviour Research and Therapy. 1983;21(3):247-51.
9. Young J, Klosko J, Weishaar M. Schema therapy: A practitioner's guide. New York: Guilford; 2006.
10. Krpan KM, Kross E, Berman MG, Deldin PJ, Askren MK, Jonides J. An everyday activity as a treatment for depression: the benefits of expressive writing for people diagnosed with major depressive disorder. J Affect Disord. 2013;150(3):1148-51.
11. Kvam S, Kleppe CL, Nordhus IH, Hovland A. Exercise as a treatment for depression: A meta-analysis. J Affect Disord. 2016;202:67-86.
12. Aylett E, Small N, Bower P. Exercise in the treatment of clinical anxiety in general practice - a systematic review and meta-analysis. BMC Health Serv Res. 2018;18(1):559-.
